# Th17 Mediated Alloreactivity Is Facilitated by the Pre-Transplant Microbial Burden of the Recipient

**DOI:** 10.1155/2012/960280

**Published:** 2012-10-09

**Authors:** Aleksandra Klimczak, Andrzej Lange

**Affiliations:** ^1^Department of Clinical Immunology, L. Hirszfeld Institute of Immunology and Experimental Therapy, Polish Academy of Sciences, 12 Rudolfa Weigla Street, 53-114 Wroclaw, Poland; ^2^Lower Silesian Center for Cellular Transplantation, National Bone Marrow Donor Registry, Grabiszyńska 105, 53-439 Wroclaw, Poland

## Abstract

Acute graft-versus-host disease (aGvHD) is a major complication after hematopoietic stem cell transplantation (HSCT) and severity of aGvHD is associated with biological and genetic factors related to donors and recipients. Studies on inflammatory pathways involved in aGvHD have shown a significant impact of the gut microflora on aGvHD development giving increasing evidence in the understanding of the response of innate and adaptive immunity to microbial products. Cytokine deregulation may increase or reduce the risk of aGvHD. Damage of tissues affected by aGvHD reflects the immunological cascade of events in this disease.

## 1. Introduction

Allogeneic hematopoietic stem cell transplantation (HSCT) is a clinically accepted procedure in some hematological malignances, aplastic anemia, and inborn errors. It is rather a complex procedure, associated with both the adverse effect aGvHD and with the presence of beneficial alloreactivity, as it is graft versus leukemia or versus cells with inborn error reaction [1–4]. Alloreactivity influences both hematological and immunological recovery. Both alloreactivity and recovery of blood cells take place in an environment full of microbial agents in a latent form or colonizing/invading the host. Innate and adaptive immunity competence prior to and after HSCT secure an event-free course after HSCT with respect to that.

### 1.1. Biology of Acute GvHD

Damage of the gastrointestinal tract during the acute phase of GvHD plays a major pathophysiological role in the amplification of this systemic disease. Several experimental and clinical observations highlight the role of effector cells of the immune system migration into the skin and gastrointestinal tract in the pathobiology of aGvHD [5]. Mice are the most often used animal model of GvHD. Differences in age, sex, genetic matching, and also gut microbiota of the mice are found to be the main players in pathophysiology of GvHD [6].

One of the first reports describing the microbial environment of the recipient as an important cofactor of gut aGvHD development was presented by Van Bekkum et al. [7, 8]. In their studies they compared the fate of conventionally and germ-free housed mice after whole-body irradiation and MHC incompatible bone marrow cell transplantation. Enteric aGvHD was less frequent in germ-free mice and in mice receiving antibiotic prophylaxis as compared to conventionally transplanted animals. The authors concluded that antigenic epitopes of microorganisms shared with gut epithelial cells may promote alloreactivity. These observations indicated that lymphocytes sensitized against microbial antigens may cross-react with epithelial cells in the gut, promoting aGvHD. Experimental studies demonstrated that loss of integrity of the gastrointestinal tract plays a major role in experimental GvHD [9]. Intestinal microflora, their antigenic challenge, and released endotoxins constitute part of the microenvironment and can serve as potent triggers of inflammation in GvHD [9].

### 1.2. Innate Immunity and aGvHD

The studies on the role of gut microflora in initiation of aGvHD help in understanding the role of the innate and adaptive immune response evoked by microbial products in this disease [10]. Conditioning regimen damage of the gut and concomitant release of endotoxins and lipopolysaccharides (LPS) from colonizing the gut microbes activate innate immunity via Toll-like receptors (TLRs), which starts a cascade of events leading to cytokine storm, which constitutes part of the aGvHD pathomechanism [9, 11, 12]. Ligation of intestinal TLR9 by bacterial DNA increases the risk of aGvHD. TLR9 knockout mice have aGvHD of a reduced activity and intestinal damage [11]. The impact of bacterial sensing via TLRs in gut aGvHD was analyzed in an intestinal mice model which shows that MyD88 (myeloid differentiation primary-response protein 88)-dependent TLR9 signaling of bacterial DNA is essential for induction of apoptosis and cell infiltrations in the gut during aGvHD [13]. Indeed, the use of oligonucleotide (iODN) 2088, which inhibits TLR9 activation in vitro, ameliorates the symptoms of gut aGvHD in mice [13]. In contrast, mutations in TLR4 (which encodes LPS receptor) have been shown to be a biological factor reducing the risk of GvHD in experimental studies [14]. 

Manipulation with gut microflora in favor of *Lactobacillus rhamnosus* GG [15] makes aGvHD less aggressive. Very recent experimental and clinical studies demonstrated that microbial chaos early after HSCT and loss of intestinal flora diversity are a potential risk factors for subsequent aGvHD development [16]. In clinical practice intestinal bacterial decontamination with metronidazole and ciprofloxacin significantly reduces the severity of gut aGvHD [17], which supports the notion that intestinal microflora play a role in the pathogenesis of aGvHD. 

The NOD2/CARD15 protein, restricted to intestinal epithelial cells and monocyte/macrophage lineage [18], plays a role in the innate immune response to bacterial infections in the gastrointestinal tract. It is at present known that NOD2/CARD15 gene mutations found in patients undergoing HSCT make them more susceptible to aGvHD [19]. The cumulative incidence of 1-year transplant-related mortality and the prevalence of severe gut aGvHD affected 49% of patients with NOD2/CARD15 gene mutation as compared to 20% incidence in those without NOD2/CARD15 gene mutation. If the mutation affects donors this proportion increases to 59% and to 83% if both recipient and donor have the gene mutated [20]. Our observations also show that NOD2/CARD15 gene mutation is associated with susceptibility to severe GvHD grade III-IV [21]. Moreover, we found that mutations in the NOD2/CARD15 gene influences the level of Th17 in blood in such a way that patients with NOD2/CARD15 mutations had lower blood values of Th17 at the time of hematological recovery in the aGvHD group [22]. 

## 2. Pathophysiology of aGvHD

**Figure 1:**
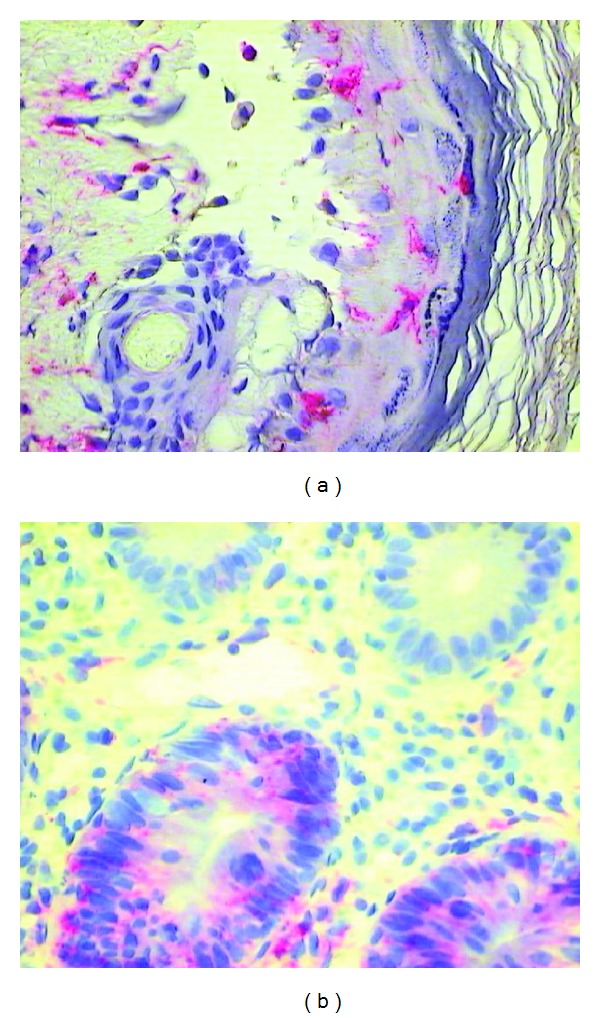
(a) HLA-DR expression on antigen presenting cells in the epidermis of the skin (+60 days after HSCT) and (b) HLA-DR expression on colon epithelial cells (+33 days after HSCT) affected by aGvHD (red staining with Permanent Red, magnifications 400x).

**Figure 2:**
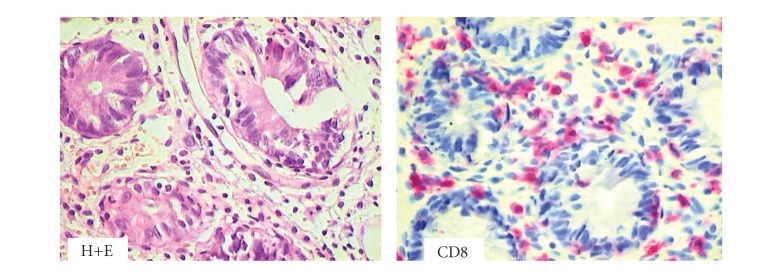
Colon biopsy specimen harvested at 33 days after HSCT from patient with clinical symptoms of aGvHD. Hematoxylin and eosin (H+E) staining documented destruction of colon crypts, and immunocytochemistry illustrate CD8+ cells invading damaged crypt epithelium (H+E magnification 200x, red staining with Permanent Red, magnifications 400x).

The conditioning regimen causes tissue damage and as a consequence several proinflammatory cytokines including IL-1 and TNF-*α*, and a set of chemokines, CCL2-5 and CXCL9-11, are released, thereby increasing expression of adhesion molecules, MHC antigens and costimulatory molecules on the host antigen presenting cells (APC) [1, 23]. Host APC, which survive the conditioning regimen damage, become activated and capable of confronting the transplant material antigens (Figure 1). Activation of donor T cells after interaction with host APC leads to their proliferation, differentiation, and migration. In the subsequent effector phase mononuclear cells invade the target tissue and accumulation of these cells leads to tissue destruction (Figure 2) [23, 24].

It is known, also from our own experience, that anti-CD52 monoclonal antibody (MoAb) (Campath-1H), if used as part of the conditioning regimen, greatly decreases the risk of aGvHD (unpublished). Anti-CD52 MoAb has a unique property to destroy not only lymphocytes but also APC [25]. 

In afferent phase 1 of aGvHD LPS of Gram-negative bacteria are the main stimulators of proinflammatory cytokines and chemokine receptors. The intensive conditioning regimen induces apoptosis and consequently epithelial cell damage, allowing LPS to enter the systemic circulation, activating host APC, which facilitates alloreactivity, leading to aGvHD [9, 26].

Activated T cells proliferate and secrete cytokines [1]. Th1 cells contribute to the cytokine storm associated with aGvHD, while Th2 cytokines may mitigate the impetus of alloreactivity [6]. Indeed, in our early studies we confirmed the presence of IFN*γ*, IP-10 as well as TNF-*α* and IL-6 transcripts in skin affected by aGvHD [27]. IL-2 and IFN*γ* prime mononuclear phagocytes to produce IL-1 and TNF-*α*. TNF-*α*, a powerful inducer of APC in the first phase, activates T cells also in the second phase of aGvHD. Again, the microbial impact plays a role in establishing a vicious circle of infection (TNF*α*, IL-6) and thus aggravation of aGvHD. 

The role of microbial infection in aggravating aGvHD has a long history [28]. Recently we added some more information as to the role of Th17+ lymphocytes, whose differentiation is strongly supported by microbial invasion. It is a step-by-step process starting with TNF-*α* and IL-1 secretion in phase 1 in response to the conditioning regimen and microbial background. Among proinflammatory cytokines, IL-6 plays an important role in aggravation of aGvHD, especially when the gut is targeted [27, 29]. This cytokine is released and generated during inflammatory processes associated with (i) the conditioning regimen, (ii) the alloreactivity associated inflammation, and (iii) bacterial and fungal infections [27]. C-reactive protein (CRP) is a reading protein of IL-6 and usually reflects microbial invasion. Notably, increase of the serum CRP level may herald gut manifestation of aGvHD [27]. Increase in serum level of IL-6 is seen early after transplant as a result of a cytokine storm described in allogeneic HSCT patients at the period of neutropenia, and then an increase may be again seen during prolonged leucopenia and at that time is usually associated with infectious complications [29]. All these events are responsible for elevation of serum CRP level during the period after HSCT [29, 30]. 

**Figure 3:**
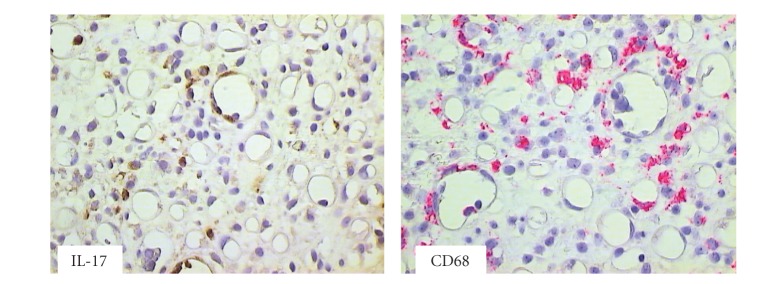
Colon biopsy specimen harvested at day 63 after HSCT from patient with clinical symptoms of aGvHD. IL-17 producing cells and macrophages CD68+ were seen within cellular infiltrates (brown staining with diaminobenzidine-tetrahydrochloride (DAB) and red staining with Permanent Red, magnifications 400x).

Following more recent observations it is known that the differentiation process of CD4+ cells into subsets depends on the cytokine milieu in their environment [31]. IL-6, if present, facilitates differentiation of CD4+ cells into Th17 cells [32, 33]. IL-17 is a cytokine of the strongest proinflammatory potential. It is known that differentiation of lymphocytes into Th17 cells may take place in the gut, where microbial products provide strong stimulation for local IL-6 production [34]. Therefore, it is not surprising that in intestinal aGvHD IL-17 producing cells are present among those infiltrating affected tissue (Figure 3) [35]. Local IL-17 production in the gut during aGvHD is seen in patients with extensive diarrhea resulting from profound damage of intestinal epithelium.

Th17 differentiation is guided by IL-6, which constitutes a primary response to bacterial and fungal infections. Th17 cells have as a hallmark receptor CCR6, which in response to their ligand CCL20 (also known as macrophage inflammatory protein-3*α*, MIP-3*α*), produced by activated macrophages in the inflammatory area of the gut, facilitates colonization of gut epithelium by IL-17 producing cells, causing severe inflammation [36, 37]. A correlation between the number of Th17 cells and the clinical course of aGvHD supports the notion that Th17 cells are involved in the active phases of aGvHD [38]. Our studies showed that IL-17 producing CD4+ lymphocytes are at a higher proportion in blood prior to aGvHD manifestation and then decrease at the time of full blown aGvHD [39]. These cells are likely marginalized in the affected tissue, exerting their strong pro-inflammatory activity. 

In conclusion, the data collected since the pioneering work of Van Bekkum strongly suggest that microbial products influence the risk of aGvHD in all phases of pathobiology of this complication via activation of APC then inducing the local production of IL-6 exemplified by CRP serum level elevation to the effector phase exerted by lymphocytes of Th17 cell characteristics. Pre- and peritransplantation colonization of recipients with bacterial and fungal germs promotes alloreactivity; therefore, microbial surveillance plays an important role in securing an event-free posttransplant course. Bacterial and fungal colonization after transplant involves both Gram-positive and Gram-negative bacteria. However, up to date is not sufficiently defined which microbial populations may exert or protect aGvHD-associated damage and inflammation because both Gram-positive and Gram-negative bacteria may overgrow the intestinal flora and may worse aGvHD [16, 28]. Therefore, both Gram-positive and Gram-negative bacteria can play a role in activation of both innate and adaptive immunity with production of IL-6 with following consequences of the presence of this cytokine which may facilitate pathomechanism of aGvHD.
